# The effectiveness of cardiac telerehabilitation in comparison to centre-based cardiac rehabilitation programmes: A literature review

**DOI:** 10.1177/1357633X221085865

**Published:** 2022-04-04

**Authors:** Olivia Owen, Veronica O’Carroll

**Affiliations:** School of Medicine, 12187 University of St Andrews, St Andrews, Scotland

**Keywords:** literatire review, cardiology, telerehabilitation, telemedicine, ehealth, telehealth

## Abstract

**Introduction:**

Cardiac rehabilitation (CR) is an effective, yet under-utilised, form of secondary prevention in cardiac patients. Telemedicine is one method of overcoming barriers to accessing CR. Previous systematic reviews highlight variation in the effectiveness of telerehabilitation programmes and current literature lacks identification of which telemedicine interventions are most effective, despite differences in the results of primary studies. The objectives of this literature review were to: evaluate the effectiveness of cardiac telerehabilitation compared to centre-based programmes for managing cardiac risk factors, satisfaction and adherence in cardiac patients; identify the technologies used to deliver CR; identify the key components of effective interventions.

**Methods:**

A literature search was conducted using MEDLINE, EMBASE and Scopus. Randomised controlled trials (RCTs) involving an intervention group that received telerehabilitation and a control group that attended a CR centre were included.

**Results:**

Twelve RCTs met the inclusion criteria. There is evidence to suggest that telerehabilitation programmes have similar effectiveness to centre-based CR. Phones were the most commonly used technology. Most studies used a combination of technologies including personal computers and self-monitoring equipment. Phase III telerehabilitation programmes using self-monitoring, motivational feedback and education were more effective than centre-based CR for increasing physical activity and functional capacity.

**Conclusion:**

Cardiac telerehabilitation is delivered by a range of technologies and has a similar effectiveness to centre-based programmes. While evidence suggests that additional health benefits are seen in patients who receive a telemedicine intervention in Phase III of CR, further evidence would be required to confidently draw this conclusion.

## Introduction

The global prevalence of cardiovascular disease (CVD) almost doubled between 1990 and 2019;^1^ patients with these conditions are at increased risk of future cardiac events^2,3^ which places a significant demand on health services.^4,5^ Cardiac rehabilitation (CR) is an evidence-based multiprofessional intervention used for secondary prevention in patients with cardiovascular disease,^6^ playing a role in preventing further cardiac events and in the re-enablement of patients.^7^ Namely, the main components of CR are exercise, education, psychosocial counselling and risk-factor management.^4,6,8–11^

Patients who undertake CR have reduced hospitalisation and mortality, alongside improved Health Related Quality of Life (HRQoL), compared with those who do not.^12,13^ Additionally, there are beneficial effects on patients’ cardiovascular risk factors, which satisfies the goal of cardiac rehabilitation. The UK-based *National Audit of Cardiac Rehabilitation* (NACR) reports that CR participation is associated with increased physical activity levels; reductions in anxiety, depression, total cholesterol (TC) and low-density lipoprotein (LDL) cholesterol levels. Furthermore, small beneficial effects are seen in the Body Mass Index (BMI), blood pressure (BP), waist circumference (WC) and alcohol consumption of patients who take part in CR.^14^ Most commonly, CR programmes are centre-based whereby patients work in groups with a physiotherapist.^14^

Despite the high burden of CVD^1–5,15^ and evidence displaying the benefits of CR participation^,12–14^ CR programmes are characterised by low rates of uptake and adherence amongst eligible patients.^14,16^ In the UK, only 50% of eligible patients partake in CR and there are concerns that the current method of delivery makes it unrealistic for goals of higher participation to be met.^14,17,18^ Some of the reasons for non-participation are geographical barriers^,19–22^ work and family commitments, and preferences for home-based exercise.^21,23^ Using telemedicine in CR, or cardiac telerehabilitation, is one way in which these barriers can be overcome.^24,25^

Previous research has reported that home-based CR programmes, including those delivered by means other than technology, were as effective as centre-based programmes for improving patients’ functional exercise capacity and quality of life.^26,27^ Regarding the use of telemedicine in CR, guidelines state that it can be considered as an option for some patients.^4,9^ However, there are inconsistencies within the evidence that was used to create these guidelines. Two systematic reviews published in 2015 reported differing findings in the research with regards to how effective telerehabilitation was in managing cardiac risk factors, particularly pertaining to the effectiveness of telerehabilitation in improving patients’ functional exercise capacity, HRQoL, anxiety and depression.^28,29^ The range of telemedicine interventions used in CR programmes could account for some of the variation in effectiveness.^28,29^ Understanding which aspects of the interventions are associated with the greatest benefit for patients could help to gain a clearer understanding of how telerehabilitation programmes could be delivered effectively. However, this has not been analysed in the current literature, despite the continued presence of discrepancies between the results of individual primary studies.^30^ These factors warrant a review of the more recent literature which will be representative of current attitudes to, as well as current technologies that are used within cardiac rehabilitation. Furthermore, the increase in the use of telemedicine in recent years, and especially during the Covid-19 pandemic means that confidence in its effectiveness is imperative.^25^

This literature review aims to appraise the most recent research that reports on the effectiveness of cardiac telerehabilitation interventions in managing the modifiable cardiac risk factors of eligible patients, as well as their satisfaction and attendance levels, compared with traditional centre-based CR. It aims to identify which technologies are used in these programmes, as well as the common features of the interventions that are most effective. This review will not include video-gaming and virtual reality (VR) as digital health interventions, as their use in CR has recently been explored.^31^

## Methods

### Search strategy

**Figure 1.**
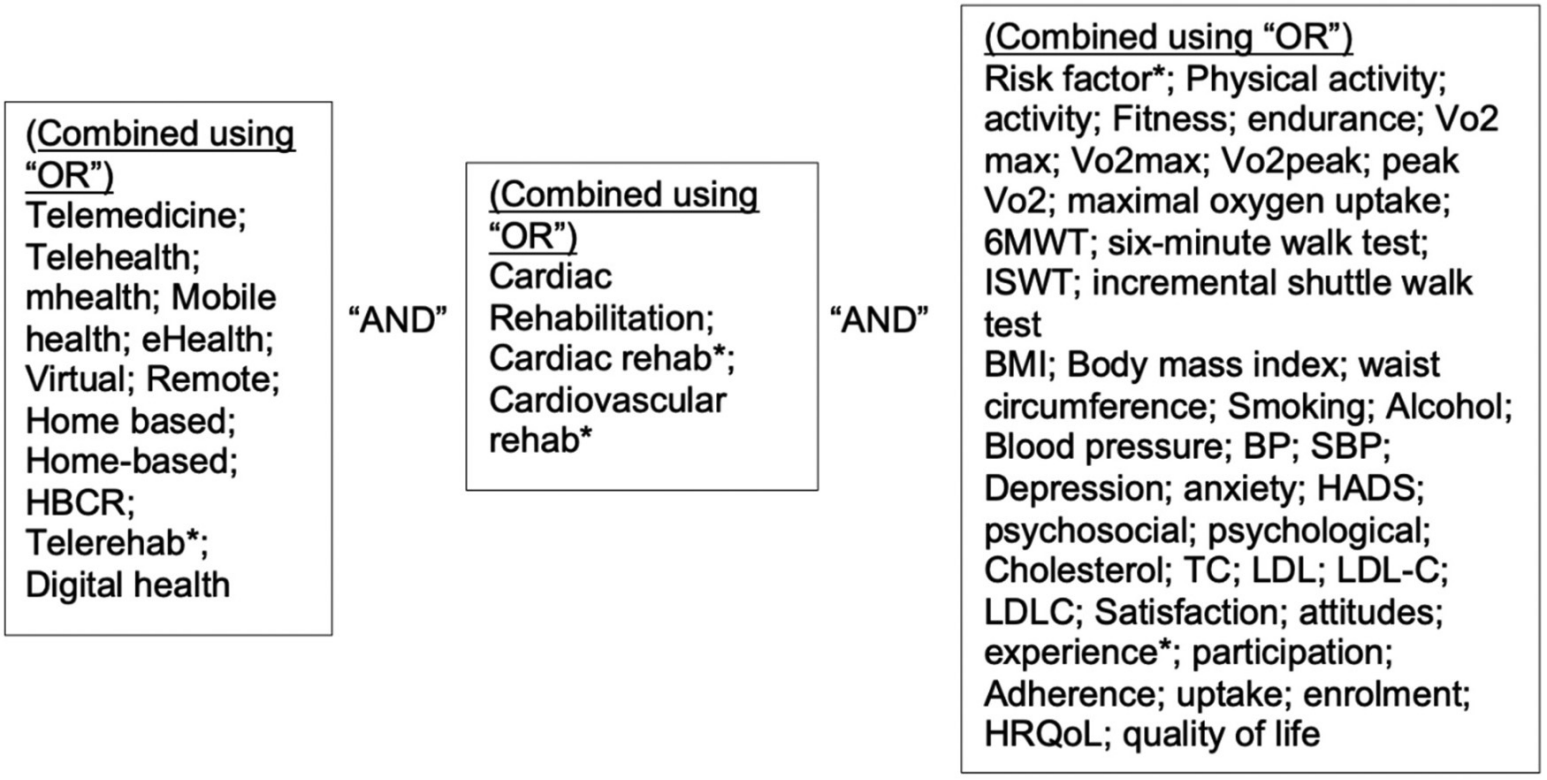
The use of boolean operators in the literature search.

A literature search was conducted using the databases *Medline*, *EMBASE* and *Scopus,* using the search terms shown in Table 1. Search terms related to outcomes were selected based on the risk factors that were reported in the latest version of the NACR.^14^ This encompassed: functional exercise capacity (FC), physical activity (PA), lipid profile, BP, BMI and WC. Search terms related to Quality of Life (QoL), satisfaction and adherence were also included as measures of acceptability of interventions to patients. Asterisks were used as truncation symbols to retrieve variations of terms. The Boolean Operators “AND” and “OR” were used to combine search terms, as shown in Figure 1.

**Table 1.** Search terms.

Part 1: Terms Related to Telemedicine	Part 2: Terms Related to CR	Part 3: Terms Related to Outcomes
Telemedicine; Telehealth; mhealth; Mobile health; eHealth; Virtual; Remote; Home based; Home-based; HBCR; Telerehab*; digital health	Cardiac Rehabilitation; Cardiac rehab*; Cardiovascular rehab*	Risk factor*; Physical activity; activity; Fitness; endurance; Vo2 max; Vo2max; Vo2peak; peak Vo2; maximal oxygen uptake; 6MWT; six-minute walk test; ISWT; incremental shuttle walk test BMI; Body mass index; waist circumference; Smoking; Alcohol; Blood pressure; BP; SBP; Depression; anxiety; HADS; psychosocial; psychological; Cholesterol; TC; LDL; LDL-C; LDLC; Satisfaction; attitudes; experience*; participation; Adherence; uptake; enrolment; HRQoL; quality of life.

**BMI:** body mass index; **HBCR**: Home based cardiac rehabilitation; **LDLC/LDL-C** = low density lipoprotein cholesterol; **TC** = total cholesterol; **VO_2_ peak/VO_2_ max **= peak/maximal oxygen uptake.

Table 2 shows the inclusion and exclusion criteria which guided the literature search. Studies available in the English language and published between 1^st^ January 2016 and 21^st^ February 2021 were included. Studies were included if they measured one or more of the outcomes listed in the NACR.^14^ Studies that measured patient satisfaction and adherence were also included. Only Randomised Controlled Trials (RCTs) with a control group that received a centre-based CR service were included because of their ability to determine cause-effect relationships and lower bias compared to other study types.^32^ Where multiple papers reported results of the same trial at different follow-up points, the paper with the longest follow-up period was used. Studies were excluded where their intervention consisted of videogaming or VR.

**Table 2.** Inclusion and exclusion criteria.

Inclusion Criteria	Exclusion Criteria
- Available in the English language. - Published in the past 5 years (Between 1st January 2016 and 21st February 2021). - Published in a peer reviewed journal - Population is eligible adults participating in cardiac rehabilitation. - The telemedicine intervention fits the definition of ‘ICT used to deliver a healthcare service’. - Must have measured the change in one or more traditional modifiable cardiac risk factor, quality of life, satisfaction, or adherence. - RCTs with a control group receiving centre-based CR.	- Studies measuring the impact of VR or video games in cardiac rehabilitation. - Studies where telemedicine is not used in the intervention group e.g. home-based phase II cardiac rehabilitation using leaflets and booklets. - Studies where the only technology used is telephone calls with a frequency of less than once per week. - Studies where the comparison group received no form of cardiac rehabilitation. - No cardiac risk factors or adherence or satisfaction are measured. - Study types which are not RCTs including systematic reviews and pilot trials. - Control group also does home-based CR rather than centre based CR.

OO completed the literature search independently. Search results from all three databases were exported to EndNote-X9. Duplicates were removed and the remaining articles were screened initially by title and abstract, followed by full text in order to determine their suitability. Reference lists of included studies were examined in order to find additional relevant studies which had not been identified through the databases. Studies were assessed for quality using the *Critical Appraisal Skills Programme* (CASP) *Checklist for Randomised Controlled Trials*^33^ and for bias using the *Cochrane* *Risk of Bias 2 (RoB 2)* tool.^34^ The included studies were categorised based on their interventions in order to allow analysis of the common features of effective interventions. The idea to categorise the interventions is from Clark et al. ^28^ However, the categories identified were reflective of the interventions that the included studies used, rather than the categories identified by Clark et al.

## Results

### Study characteristics

**Figure 2.**
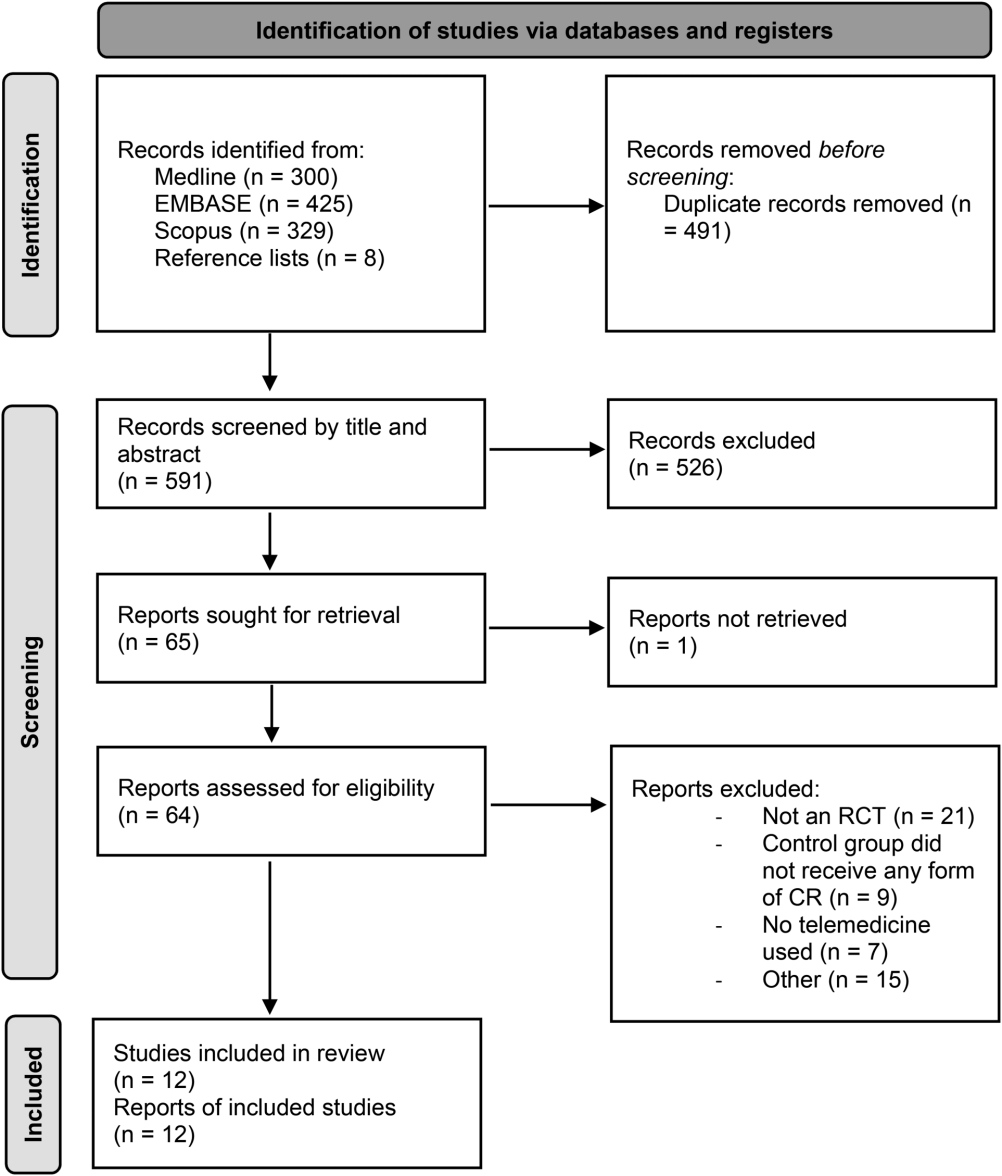
PRISMA 2020 flow diagram. *From:* Page MJ, McKenzie JE, Bossuyt PM, Boutron I, Hoffmann TC, Mulrow CD, *et al*. The PRISMA 2020 statement: An updated guideline for reporting systematic reviews. BMJ 2021;372:N71. doi: 10.1136/bmj.n71.

As shown in Figure 2, twelve RCTs met the inclusion criteria and were included in this review. One paper displayed the results of a psychological sub-study of patients in an RCT, for which the original publication did not meet the inclusion criteria due to the date published.^35^ The average sample size across all twelve RCTs at enrolment was 114.^35–46^ All studies other than the *CR4HER* trial^,36^ which was women only, included both men and women in their inclusion criteria. Across the eleven mixed-sex trials, 17% of participants whose baseline characteristics were reported were female.^35,37–46^ Seven out of twelve trials only included patients who either had a pre-defined digital literacy^35,37,38^ or internet and PC/smartphone access prior to enrolment.^39–42^ Across all twelve studies, an attrition rate of 27% was observed.^35–46^ Most studies evaluated the effects in the short term (follow up less than one year)^36–38,42–46^ but four studies had a follow up period of one year or greater.^35,39–41^

 Table 3 summarises the characteristics of all included RCTs.

**Table 3.** Characteristics of included RCTs.

Study, (Location)	Participants	Telemedicine Intervention	Comparison	Follow up	Measured Outcomes	Results
**Exercise-based Phase II Telerehabilitation**						
**Bravo-Escobar et al**., 2017^37^ (Spain)	n = 27 Patients with stable cardiomyopathy post- CABG or stent-angioplasty.	8-week home-based exercise programme. Patients attended the centre once per week and exercised at home (with remote monitoring and a smartphone application to upload information) twice per week.	8-week centre-based exercise programme. Patients attended a centre for supervised exercise 3 times per week. (All components of CR, other than exercise, were delivered in the outpatient clinic for both groups. All patients were encouraged to exercise every day).	8 weeks	Functional capacity, BP, lipids, BMI, WC, HRQoL	Functional capacity: Mean exercise time in exercise testing increased significantly in both groups (CG: + 1.4 min; IG: + 0.73 min, p = 0.03). Mean METs during exercise testing increased significantly in both groups (CG: + 1.3; IG: + 0.73, p = 0.03). There were no significant differences between groups for the change in exercise time (p = 0.32) or METs (p = 0.49). Health-Related Quality of life: Significant difference between groups in the change in SF-36 score (p = 0.004). With SF-36 score improving significantly from 53.33(19.80) to 63.63(21.00) in the control group and worsening non-significantly from 27.93(22.57) to 43.62(24.20) in the intervention group. (Results expressed as Mean (SD)) There were no significant time or between-group differences for other outcomes.
**Kraal et al**., 2017^40^ *The FIT@Home Study* (The Netherlands**)**	n = 90 Patients entering CR post- ACS or revascularisation procedure.	12-week home-based exercise programme. Patients used a chest HR monitor and web portal to upload data. Patients received weekly phone calls from physiotherapists with motivational feedback.	12-week centre-based exercise programme. (All components of CR, other than exercise, were delivered in the outpatient clinic for both groups).	1-year post-discharge	Functional capacity, PA, anxiety, depression, HRQoL, adherence, satisfaction.	Functional Capacity: Significant increase in mean VO_2 _peak in both groups (CG: + 3.5mL/min/kg, p < 0.001; IG: + 3.3mL/min/kg, p < 0.001) at follow-up, with no significant difference between groups (p = 0.865). Physical activity: There were no significant time or between-group differences for physical activity levels. Anxiety and depression: Anxiety decreased in both groups at follow-up (Change in mean SF-36 score: -1.13 in CG, p > 0.05; -1.39 in IG, p < 0.01); there were no significant changes in depression scores. There were no between-group differences for changes in anxiety and depression. Health-Related Quality of life: No significant difference in change in HRQoL over time or between groups. Adherence: Patients in the intervention group attended 2 extra sessions on average (/24) Satisfaction: Intervention group had significantly higher satisfaction rates (8.7 vs 8.1/10, p = 0.02).
**Maddison **et al**.,** 2019^43^ (New Zealand)	n = 162 Patients with a diagnosis of CHD in the past 6 months	12-week home-based exercise programme. Patients were given individualised exercise prescriptions and a smartphone application for self-monitoring, as well as real-time and post-training feedback.	12-week centre-based exercise programme. (All components of CR, other than exercise, were delivered in the outpatient clinic for both groups).	12 weeks, 24 weeks	Functional capacity, PA, BP, lipids, BMI, WC, HRQoL	Functional Capacity: Telemedicine was not inferior to centre-based exercise for VO_2_max. WC: No significant difference or p- value was reported. PA: There was a small difference in change in sedentary time in favour of the intervention group. No p-value reported. Adherence: On average, the intervention group attended 2 more sessions than the control group (out of 36 sessions prescribed) There were no significant differences reported for other outcomes.
**Batalik et al**., 2020^42^ (Czech Republic)	N = 51 Patients with CVD, post- revascularisation procedure	12-week home-based exercise programme. Patients used a wrist HR monitor and web portal to upload data. They received motivational feedback from physiotherapists via telephone.	12-week centre-based exercise programme (Both groups received the same educational booklet).	12 weeks	Functional capacity, HRQoL, adherence	Functional Capacity: Significant increase in mean VO_2 _peak in both groups but no difference between groups (CG: + 2.5 mL/kg/min, p = 0.02; IG: + 2.8 mL/kg/min, p = 0.04). Quality of Life: SF-36 scores improved significantly in both groups from pre- to post-programme with no significant difference between groups (CG: + 10.6, p = 0.01; IG: + 8.9, p = 0.01). Adherence: The intervention group attended a higher percentage of sessions (88.2 vs 83.6%, no p value) but 32% of phone calls were missed.
**Exercise and Education/Behaviour-change Based Phase II Telerehabilitation**						
**Grace et al**., 2016^36^ *The CR4HER Study* (Canada)	n = 169 Female patients who were eligible for CR.	4–6-month home-based phase II CR programme. Individualised exercise prescription and educational materials to complete at home. Patients had phone calls once or twice per week to review exercise and educational materials with a physiotherapist	4–6-month centre-based phase II CR programme with exercise and education sessions. Patients were allocated in a 1:1:1 ration to home based (HB), centre-based women only (WO) and centre-based mixed sex (MS) CR services.	6 months	Functional capacity, adherence	Functional Capacity: Per protocol analysis showed a significant increase in VO_2 _peak for all groups; with no significant difference between groups at 6 months. As-treated analysis showed no significant difference in the VO_2 _peak in the home-based group from pre-to post- programme; and that the mixed-sex centre-based group had significantly higher functional capacity than the home-based group at 6 months (MS: 19.69mL/min/kg; HB: 15.5mL/min/kg, p = 0.046). Adherence: No significant difference between all three groups in per-protocol analysis; but as-treated analysis showed higher adherence in the home-based group compared to the centre-based women only group (p < 0.05) (Mean % sessions – HB:75.32, MS: 65.51, WO: 59.94).
**Hwang et al**., 2017^44^ (Australia)	n = 53 Patients with heart failure.	12-week home-based phase II CR programme. Patients had self-monitoring equipment and used real-time videoconferencing for exercise and education sessions in groups of up to 4 patients, supervised by a physiotherapist	12-week centre-based phase II CR programme.	Functional capacity, HRQoL, adherence, satisfaction	12 weeks, 24 weeks.	Functional capacity: Significant overall improvement in mean 6MWD (CG: + 28m; IG: + 28m, p = 0.048) but no significant difference between groups (p = 0.24). IG was not inferior to CG for 6MWD at 12 weeks, but this could not be shown at 24 weeks. Quality of life: Both groups saw a significant improvement in MLWHFQ score (CG: -8, IG: -13, p value not stated), with no significant between-group difference. Adherence: The intervention group had significantly higher rates of attendance, attending 6 extra sessions on average (/24). Satisfaction: Satisfaction rates were high in both groups with no significant between group difference (Both groups had an average CSQ score of 32/32)
**Widmer et al**., 2017^45^ (USA)	n = 80 Patients who had undergone PCI for ACS.	12-week centre-based phase II CR plus access to a digital health intervention. Patients uploaded information about their exercise and diet to a web application and received educational materials and automated advice.	12-week centre-based phase II CR programme.	Functional capacity, BP, Lipids, BMI, WC, depression, HRQoL	12 weeks.	BMI and waist circumference: Significant difference between groups in decrease in BMI (-1.6 in IG, -0.8 in CG, p = 0.01) and waist circumference (-8.3cm in IG, + 1.1cm in CG, p = 0.01). Quality of life: The intervention group had a significantly greater improvement in QoL (change in mean Dartmouth QOL score – CG: -2.4; IG: -7.2, p = 0.03) P-values for time effects were not reported. No further significant differences between groups were reported.
**Spindler et al**., 2019^35^ (Denmark)	n = 151 Patients with a diagnosis of HF, artery stenosis, past CABG or valve surgery.	12-week home-based phase II CR programme. Patients used a web-application to upload self-monitoring data and receive educational materials. Patients could use the application to communicate with healthcare staff.	12-week centre-based phase II CR programme.	Anxiety, depression, HRQoL	12 weeks, 24 weeks, 1 year	Significant improvements with a small to medium effect size were seen in both groups for all 3 outcomes. (HADS Depression mean score – CG: -0.87; IG: - 1.06) (HADS Anxiety mean score – CG: -1.15; IG: -2.00) No overall score for SF-36 was reported. No significant differences between groups were reported for anxiety, depression or QoL.
**Phase III Telerehabilitation**						
**Frederix et al**., 2017^39^ *Telerehab III* (Belgium)	n = 140 Patients with CAD and/or CHD who were half-way through a 12-week centre-based CR programme.	6 months of cardiac telerehabilitation after 12 weeks of traditional centre-based CR. Patients had individualised exercise prescriptions and did self-monitoring. Patients uploaded data to a web-application and received weekly educational materials, feedback and advice.	12-week centre-based CR programme followed by a traditional phase III follow-up.	Functional capacity, PA, HRQoL, BP, lipids	24 weeks, 2 years	Functional Capacity: The intervention group had a significantly higher aerobic capacity (MD: 2.139 mL/min/kg, p = 0.032) at follow-up. PA IG had a significantly higher moderate or vigorous physical activity levels (p = 0.01) at follow up. Quality of life: There was a small overall improvement in HRQoL in the intervention group; the improvement was significantly greater than that of the control group at follow up (p = 0.005). Lipids: Significant increase in TC levels in both intervention and control groups once their treatments ended (p = 0.013, 0.016). There were no significant time or between group differences for other outcomes.
**Skobel et al**., 2017^38^ *The Heart Cycle Trial* (Germany, Britain and Spain)	n = 132 Patients with CAD who had also had an MI or acute coronary intervention. Patients had completed a phase II CR programme prior to the beginning of the trial.	Patients received usual care plus 6-months access to a web-application with exercise prescriptions and remote monitoring. Educational materials were provided via the application.	A 6-month period of usual care according to guidelines for phase III of CR.	6 months	Functional capacity, BP, lipids, BMI, anxiety, depression, HRQoL	Functional Capacity There was a statistically significant difference in the change in mean VO_2_peak between groups, in favour of the intervention group (IG: + 1.76mL/min/kg; CG: -0.4mL/min/kg). BP Statistically significant difference between groups for change in systolic BP (IG: + 6mmHg; CG: -8mmHg, p = 0.003) No time or between group differences were reported for other outcomes
**Avila et al**., 2020 ^41^ (Belgium)	n = 90 Patients with CAD who had already completed a phase II CR programme.	An additional 12-week home-based CR programme with individualised exercise prescriptions and self-monitoring. Patients uploaded data to a web-application and received telephone and e-mail feedback from physiotherapists.	Usual care, or an additional 12 weeks of centre-based CR at the end of the phase II programme (Patients were allocated on a 1:1:1 basis)	12 weeks, 1 year	Functional capacity, PA, BP, lipids, BMI, WC, HRQoL	Functional Capacity No significant time or between-group differences in VO_2 _peak. PA No between-group differences in physical activity. Quality of Life Health related QoL was high in all three groups at follow up, with no significant between- group differences. There were no significant time or between-group differences in any of the other outcomes.
**Lunde **et al**.,** 2020^46^ (Norway)	n = 113 Patients with heart disease participating in CR.	1 year of individualised telemedicine-based follow up after phase II CR. Patients accessed an application which encouraged goal setting and sent reminders to patients. Patients received motivational feedback via the application and phone calls; patients could submit questions to healthcare professionals via the application.	Usual care.	1 year	Functional capacity, PA, BP, lipids, HRQoL	Functional Capacity There was a significant between group difference in VO_2 _peak at follow up, in favour of the intervention group. (Change in mean VO_2_ peak – CG: -0.8mL/min; IG: + 1.4mL/min/kg, p < 0.001) PA IG had a higher PA level compared to the CG, completing 0.9 more exercise sessions per week (p < 0.001). BP There was a significant increase in systolic blood pressure in both groups (CG: + 9mmHg, p < 0.05; IG: + 9mmHg, p < 0.001). Lipids No significant time or between group differences for lipid profile. Other than small changes in mean HDL cholesterol levels (CG: + 0.1, p < 0.05; IG: 0 + /-0.2, p < 0.05) Quality of Life HRQoL increased in the intervention group only (mean HeartQoL score + 0.21, p < 0.05) but there was not a statistically significant difference between groups at follow up.

**ACS**: Acute Coronary Syndrome; **CABG**: Coronary Artery Bypass Graft; **CAD**: Coronary Artery Disease; **CG** = Control Group; **CHD**: Coronary Heart Disease; **CSQ**: Client Satisfaction Questionnaire; **CVD**: Cardiovascular Disease; **HF**: Heart Failure; **IG** = Intervention Group; **MD**: Mean Difference; **MLWHFQ**: Minnesota Living With Heart Failure Questionnaire; **METs**: Metabolic Equivalents; **PCI**: Percutaneous Coronary Intervention; **SF-36**: Short-Form 36; **VO_2_peak**: Peak Oxygen Uptake.

### Telerehabilitation and interventions

Seven studies^35,36,39,40,42,43,46^ used telemedicine as a replacement of one or more aspects of care. Five studies^37,38,41,44,45^ used telemedicine as an adjunct to usual care. Specific details of the interventions used in individual trials are summarised in Table 3. Table 4 shows the technologies that were used to deliver CR in each study.

**Table 4.** Technologies used for cardiac rehabilitation in included studies.

Study	Technologies used	Use of phone and/or PC
**Grace et al**. 2016^36^	Telephone	Phone calls
**Bravo-Escobar et al**. 2017^37^	ECG sensor, smartphone	Smartphone application
**Frederix et al**., 2017^39^	Accelerometer, PC, mobile phone	Web-page access, SMS text messages, e-mails
**Hwang et al**., 2017^44^	PC, mobile phone, automatic sphygmomanometer, finger pulse oximeter	Videoconferencing, phone calls used for technical enquiries
**Kraal et al**., 2017^40^	Chest-worn HR sensor, PC, telephone	Web application, phone calls
**Widmer et al**., 2017^45^	Accelerometer, PC, mobile phone	Web-application; SMS text messages
**Skobel et al**., 2017^38^	ECG sensor, smartphone, PC	Smartphone application, web application (for patient/health worker use respectively)
**Maddison et al**., 2019^43^	Chest-worn sensor (accelerometery, ECG, HR, RR), smartphone or PC	Smartphone- or web-application
**Spindler et al**., 2019^35^	Pedometer, HR monitor, sphygmomanometer, PC	Web-application
**Avila et al**., 2020^41^	PC, telephone	Web-application, e-mails, phone calls
**Batalik et al**., 2020^42^	Wrist heart rate monitor, PC, telephone	Web-application, phone calls
**Lunde et al**., 2020^46^	Smartphone	Smartphone application

**ECG**: Electrocardiogram; **HR**: Heart Rate; **PC**: Personal Computer; **SMS**: Short Message Service.

In the included studies, CR was considered in the following three phases: (I) Rehabilitation services that begin when a patient is in hospital; (II) Outpatient CR programmes; (III) Long-term follow up and maintenance.^35–46^ The telerehabilitation interventions that were investigated in the included trials are organised into three categories:

1. Exercise-based phase II telerehabilitation.^37,40,42,43^
2. Exercise and education/behaviour-change based phase II telerehabilitation.^35,36,44,45^
3. Phase III telerehabilitation.^38,39,41,46^

### Functional capacity (FC)

Eleven studies measured FC.^36–46^ Six studies reported no significant between-group differences in FC.^37,40–43,45^ Three studies found significant differences in favour of the telerehabilitation group.^38,39–46^ Grace et al. found, in an ‘as-treated’ analysis, that the home-based group had a lower FC than the mixed-sex centre-based group (p = 0.046).^36^ Hwang et al. could not conclude that TR was not inferior to centre-based CR.^44^

### Physical activity levels (PA)

Five studies measured PA.^39–41,43,46^ Two reported that there were no differences between groups.^40,41^ Three studies reported a significant difference in favour of the intervention group.^39,43–46^

### Blood pressure (BP) and lipids

Seven studies measured BP and lipids.^37–39,41,43,45,46^ Four studies did not report time or between-group differences for these outcomes.^37,41,43,45^ Skobel et al. reported that BP increased in the intervention group and decreased in the control group.^38^ Frederix et al., reported that total cholesterol (TC) increased in both groups during the follow- up period.^39^ Lunde et al. found that there was an increase in systolic BP and small changes in HDL cholesterol in both groups.^46^

### BMI and waist circumference

Four studies included BMI and waist circumference as outcomes.^37,41,43,45^ Additionally, Skobel et al. reported patients’ BMI only as an outcome.^38^

Four studies found no significant time or between-group differences in BMI and waist circumference.^37,38,41,42^ Widmer et al. found that the intervention group had significantly greater decrease in BMI and waist circumference than the control group.^45^

### Anxiety and depression

Three studies reported anxiety and depression as outcomes^35,38,40^ and Widmer et al. reported depression.^45^ No significant differences between groups were reported.^35,38,40,45^ Kraal et al. found that anxiety decreased in both groups.^40^ Spindler et. al found that anxiety and depression decreased in both groups^35^ and Skobel et al. found no significant changes over time.^38^ Widmer et al. did not report significance of changes over time.^45^

### Health related *quality of life*

Eleven studies reported HRQoL.^35,37–46^

Three studies reported significant differences between groups. Bravo-Escobar et al. reported improvements in the control group (p = 0.007), and non-significant deterioration in the intervention group.^37^ Frederix et. al and Widmer et. al found that there was a greater improvement in quality of life in the intervention group than the control group (p = 0.005 and 0.003, respectively).^39,45^

Of the eight studies that reported no significant differences between groups, four reported that QoL improved^35,41,42,44^ and two reported that it did not.^38,40^ Lunde et al. reported that QoL improved in the intervention group, but between-group differences were insignificant.^46^ Widmer et al. did not report significance of changes over time.^43^

### Adherence

Five studies which measured adherence found that it was higher in the intervention group.^36,40,42–44^ Two studies reported that the higher value in the range of number of sessions attended was greater than the total number of sessions prescribed in the intervention group.^40,42^ Lunde et al. measured adherence in the intervention group only, finding that it was ‘high’, with 71% of patients completing all tasks.^46^

### Satisfaction

Two studies measured satisfaction as outcomes.^40,44^ Hwang et al. reported equally high satisfaction in both treatment groups.^44^ Kraal et al. reported higher satisfaction in the intervention group.^40^

### Cigarette and alcohol consumption

No included studies measured cigarette and alcohol consumption as outcomes.

### Assessment of quality and bias

Table 5 shows the results of individual studies from the CASP RCT checklist Table 6 presents a summary of risk of bias in each study.

**Table 5.** Results from the CASP RCT checklist for included studies.

	**Question**												
Study	**1**	**2**	**3**	**4**			**5**	**6**	**7**	**8**	**9**	**10**	**11**
				**i**	**ii**	**iii**							
**Grace et al**. 2016^36^	Y	Y	N	N	N	Y	Y	N	Y	Y	Y	N	CT
**Bravo-Escobar et al**. 2017^37^	Y	Y	Y	N	N	CT	N	Y	Y	Y	CT	N	N
**Frederix et al**. 2017^39^	Y	Y	Y	N	N	Y	Y	Y	Y	Y	Y	CT	Y
**Hwang et al**. 2017^44^	Y	CT	Y	N	N	Y	N	Y	Y	Y	Y	CT	Y
**Kraal et al**. 2017^40^	Y	Y	Y	N	N	N	CT	Y	Y	Y	Y	CT	Y
**Widmer et al**. 2017^45^	Y	Y	Y	N	N	Y	Y	Y	Y	Y	CT	CT	N
**Skobel et al**. 2017^38^	Y	Y	Y	N	N	Y	CT	CT	Y	Y	CT	N	N
**Maddison et al**. 2019^43^	Y	Y	Y	N	N	Y	Y	Y	N	Y	Y	CT	Y
**Spindler et al**. 2019^35^	Y	Y	Y	N	N	CT	Y	Y	Y	Y	CT	CT	CT
**Avila et al**. 2020^41^	Y	Y	Y	N	N	Y	CT	Y	Y	Y	CT	Y	Y
**Batalik et al**. 2020^42^	Y	Y	Y	N	N	Y	Y	Y	Y	Y	CT	N	Y
**Lunde et al**. 2020^46^	Y	Y	Y	N	N	Y	Y	Y	CT	Y	CT	Y	Y

(Y = Yes, N = No, CT = Can’t tell)

**Table 6.** Overall risk of bias in included studies.

Study	Domain 1: *Bias arising from the randomisation process*	Domain 2: *Bias due to deviations from intended interventions*	Domain 3: *Bias due to missing outcome data*	Domain 4: *Bias in measurements of outcomes*	Domain 5: *Bias in selection of the reported result*	
**Grace et al**. 2016^36^	L	H	SC	L	L	
**Bravo-Escobar et al**. 2017^37^	SC	SC	L	SC	L	
**Frederix et al**. 2017^39^	L	SC	L	SC	L	
**Hwang et al**. 2017^44^	L	SC	L	SC	L	
**Kraal et al**. 2017^40^	L	SC	SC	SC	L	
**Widmer et al**. 2017^45^	SC	H	L	SC	L	
**Skobel et al**. 2017^38^	L	H	H	SC	L	
**Maddison et al**. 2019^43^	L	SC	L	SC	L	
**Spindler et al**. 2019^35^	L	SC	L	SC	L	
**Avila et al**. 2020^41^	L	SC	SC	SC	L	
**Batalik et al**. 2020^42^	L	SC	L	SC	L	
**Lunde et al**. 2020^46^	L	SC	L	L	L	

L = Low; SC = Some Concerns; H = High.

## Discussion

### Technologies used to deliver cardiac telerehabilitation

This review highlighted that CR can be delivered through a range of technologies; a finding that is consistent with a recent systematic review.^30^ Phones were the most commonly implemented technology which were used for mobile applications, phone-calls or text messages. Most studies also used remote self-monitoring equipment,^35,37–39,40,42–45^ which has previously been reported to enhance patients’ motivation for behaviour change.^47,48^ PCs for web- applications, videoconferencing and e-mailing were other examples of technologies that were also used. Despite patients valuing the social aspect of attending CR-centres^,49^ none of the studies (except for Hwang et al. who used videoconferencing for group-exercise sessions) used telemedicine to incorporate a social or peer-support aspect into the programmes.^35–46^

In relation to the technologies used, these findings differ from the findings of a systematic review conducted by Huang et al. in 2015.^29^ This previous review reported that out of nine studies, seven used phone calls as their only form of technology. The remaining two studies were reported to have used a computer application alongside phone calls with remote monitoring. This may be more reflective of the technologies that were used over 10 years ago, as the review by Huang et al. only considered studies that had been published prior to 2011.^29^

### The effectiveness of cardiac telerehabilitation

Overall, the results showed that telerehabilitation had a similar overall effectiveness as centre-based CR in managing the outcomes that this review aimed to evaluate.^35–46^ The results also showed that CR attendance is slightly higher when patients used telerehabilitation services compared to centre-based CR.^36,40,42–44^ This suggests that telemedicine can be used to effectively deliver CR services, either to complement centre-based services or, for some patients, replace them.^35–46^ This is an important finding, particularly in view of the increased use of technology to increase the efficiency of healthcare,^24,25^ the impact of Covid-19 on in-person consultations^50^ and previous uncertainties regarding the effectiveness of the use of telemedicine in CR.^28,29^ Since many of the RCTs in this review enrolled patients who had a pre-defined digital literacy or ownership of certain technologies, there is no evidence that telerehabilitation is an appropriate replacement to centre-based CR for all patients.^35,37–42^ This is in keeping with the *World Health Organization* recommendation that telemedicine is used in order to complement existing services, rather than to replace them.^25^

For BP, lipids, BMI, waist circumference, anxiety, depression and quality of life, the studies included in this review produced varying results with regards to the overall effectiveness of CR in general in improving these outcomes.^35,37–46^ This contradicts previous findings where CR was reported to be an effective form of secondary prevention and positively impacted these outcomes.^13,14^ A reason for this difference in findings could be due to the small number of studies measuring these outcomes in this review.

### Common features of the most effective telerehabilitation programmes

Waist circumference, adherence, satisfaction, anxiety and depression were measured by few studies as outcomes (five, two, three and four studies measured these outcomes, respectively).^35–46^ As such, identifying common components of interventions that were most effective in managing these outcomes was not possible. A larger number of studies reported physical activity, functional capacity, quality of life, blood pressure and lipids.^35–46^ The studies reporting physical activity and functional capacity^35–46^ were consistent in showing that the most effective interventions were delivered in Phase III of CR and included strategies which target behaviour change. These strategies were: self-monitoring, education and feedback based on the principles of motivational interviewing.^38,39–46^ This is contrary to the results of a RCT by Snoek et al.^51^ that was not included in this review, where the intervention group similarly received a telemedicine intervention in phase III of CR, whereby healthcare providers were also trained in motivational interviewing. Snoek et al. found no significant differences between groups for changes in functional capacity and physical activity levels, highlighting that additional evidence may be necessary to draw this conclusion confidently.^51^

In 2015, Clark et al. reported that telerehabilitation programmes that were most effective were those that were individualised.^28^ As reported in Table 3, individualised programmes were used in several studies across all categories^35,36,39,41,43,46^ and were common in Phase III programmes, which could be another reason for their effectiveness. However, studies in categories other than Phase III Telerehabilitation that used individualised programmes did not report an overall higher effectiveness compared to usual care.^35,36–43^ Guidelines state that all CR programmes should be individualised^4,7^ and this may have been a feature of more studies without it being stated. Clark et al.^28^ also found that exercise-based telerehabilitation programmes were not an effective alternative to usual care. However, the results of this review did not suggest that Exercise-based Phase II CR programmes were less effective than those that included both exercise and education.^35–37,40,42–45^ Patients in these studies received an educational component of CR delivered through means other than technology, as per guidance, which could be one reason for the difference in results.^37,40,42,43^

### Summary of limitations of reviewed studies

The generalisability of these results is limited firstly by the proportion of female patients in the included studies, which is an under-representation of the population of women that are eligible for CR.^14,15,35–46^ Secondly, few studies used a true intention-to-treat analysis, which is compounded by high attrition rates throughout the studies and an overall attrition rate of 27%.^35–46^ In studies by Grace et al. and Skobel et al., concerns arose when patients listed the telerehabilitation intervention itself as a reason for leaving the trial, highlighting the importance of accessibility in the technologies used in healthcare.^36,38^

None of the included studies measured all the outcomes specified by the NACR.^14^ In particular, smoking and alcohol consumption were not measured as outcomes by any of the studies despite their role in cardiovascular disease^35–46,52^ and the recommendations in guidance for these behaviours to be addressed in CR programmes.^4^ Moreover, since the follow up period of studies was mostly under one year,^36–38,42–45^ additional evidence would be required to confidently conclude that these interventions are effective in managing all cardiac risk factors and that the results remain present long-term.

### Limitations of the review methodology

This literature review was undertaken as part of a programme of academic study by the main author. The selection of studies, data extraction and appraisal of studies were undertaken independently by the main author (OO).

Although RCTs are suitable for minimising bias and determining a cause-effect relationship, many otherwise-eligible studies may have been excluded from this review due to their study design. For example, one non-randomsied study had similar fidings to that of this review, reporting no significant differences between the telemedicine and control groups in the change in patients' cardiovascular risk factors over time.^53^ Cost and adverse events were not considered as outcomes in this review. However, results from an economic analysis of a RCT show that a phase II telerehabiliation programme was associated with a small reduction in cost for both the patient and healthcare provider compared to a centre based programme, where patients in both groups saw similar improvements in health outcomes (this included BMI, WC, waist circumference depression, anxiety and functional capacity). Researchers also identified the potential for further cost savings if the telerehabiliation programme was to be implemented on a larger scale.^54^

## Conclusion

The main findings of this literature review are that cardiac telerehabilitation can be delivered through a range of technologies and that it has a similar effectiveness to traditional centre-based programmes in patients for whom the use of technology is appropriate. The interventions which appeared to be most effective for managing patients’ functional capacity and physical activity were typically Phase III interventions, which included motivational feedback, self-monitoring or educational components.^35–46^ The limitations highlighted in this review suggest that future primary research in this area should consider measuring the effectiveness of cardiac telerehabilitation on all of the relevant cardiac risk factors that CR seeks to address, especially patients’ smoking and alcohol consumption habits.

## Statement of originality

The authors confirm that this work has not been previously published in any other format, in any other publication, in whole or in part.
